# Chemical Compounds Toxic to Invertebrates Isolated from Marine Cyanobacteria of Potential Relevance to the Agricultural Industry

**DOI:** 10.3390/toxins6113058

**Published:** 2014-10-29

**Authors:** Magbubah Essack, Hanin S. Alzubaidy, Vladimir B. Bajic, John A. C. Archer

**Affiliations:** Computational Bioscience Research Center (CBRC), King Abdullah University of Science and Technology (KAUST), Thuwal 23955-6900, Jeddah, Saudi Arabia; E-Mails: magbubah.essack@kaust.edu.sa (M.E.); hanin.alzubaidy@kaust.edu.sa (H.S.A.); vladimir.bajic@kaust.edu.sa (V.B.B.)

**Keywords:** cyanobacteria, moluscicide, snail, slugs, worms, crustacean, brine shrimp, invertebrate, environmental, agriculture, climate change, toxic compounds

## Abstract

In spite of advances in invertebrate pest management, the agricultural industry is suffering from impeded pest control exacerbated by global climate changes that have altered rain patterns to favour opportunistic breeding. Thus, novel naturally derived chemical compounds toxic to both terrestrial and aquatic invertebrates are of interest, as potential pesticides. In this regard, marine cyanobacterium-derived metabolites that are toxic to both terrestrial and aquatic invertebrates continue to be a promising, but neglected, source of potential pesticides. A PubMed query combined with hand-curation of the information from retrieved articles allowed for the identification of 36 cyanobacteria-derived chemical compounds experimentally confirmed as being toxic to invertebrates. These compounds are discussed in this review.

## 1. Introduction

Changing weather patterns have rendered pest control strategies ineffective. The climate factors that aid pest invasion include increasing average temperatures, warmer winter minimum temperatures, changes in precipitation patterns and water shortages. In 2012, the Grain Research and Development Corporation (GRDC) in Australia reported that an increase in mollusc activity has caused crop damage, clogging of machinery, contaminated harvested grain and poses a threat to grain exports [1]. GRDC additionally reported that the unusually moist spring, summer and autumn seasons favour snail breeding and have triggered snails to breed opportunistically and proposed a more concerted snail control regime to be implemented across the seasons [1]. Moreover, GRDC continues to investigate alternative forms of snail control, including new biological agents. Similarly, GRDC are pursuing research into epidemiology and control of emerging invertebrate pests including armyworms, earwigs, millipedes, weevils and rutherglen bugs [2].

Current Integrated Pest Management (IPM) solutions incorporates stubble management, burning and baiting for the in-field control of snails in the four months leading up to the cropping phase that is complemented with new grain cleaning approaches [3,4]. Baiting is the only control option once crops have been sown. In its absence, the benefits of stubble management and burning may be lost. However, only mature snails (snails more than 7 mm in diameter (round) or length (conical)) feed on the bait, while juvenile snails feed primarily on decaying plants. Thus, implemented in-field controls that have minimal effect on juvenile snails or snail eggs, combined with climate changes triggering opportunistic snail breeding, have rendered current IPM solutions ineffective [4]. The active ingredients in snail bait commonly used in broadacre agriculture include Metaldehyde, Methiocarb and Iron EDTA complex. Methiocarb and Metaldehyde are non-specifically toxic to a wide range of organisms including mammals, whereas iron chelate toxicity is low and target invertebrates such as molluscs and crustacean [5]. Since, iron EDTA complex is only an attractive feed for mature snails and changing weather patterns made IPM solutions less effective and harder to implement, a more effective invertebrate-targeted biological agent is required. This invertebrate-targeting biological agent should be an attractive feed for both mature and juvenile snails, as well as an effective bio-ovicide.

Thus, chemical compounds toxic to invertebrates only are emerging as an important research area for the agricultural industry. Here, we review toxic chemical compounds isolated from cyanobacteria that may have the potential to be used as bio-molluscicides and bio-ovicides against adult and juvenile snails and its eggs. We used the National Center for Biotechnology Information (NCBI) PubMed database to search for cyanobacteria-derived compounds toxic to invertebrates using the following keywords:

(cyanobacterium OR algae OR cyanobacteria OR algal) AND (molluscicide OR snail OR snails OR slug OR slugs OR mollusc OR molluscs OR crustacean OR crustaceans OR brine shrimp OR brine shrimps OR invertebrate OR invertebrates OR worm OR worms).

**Figure 1:**
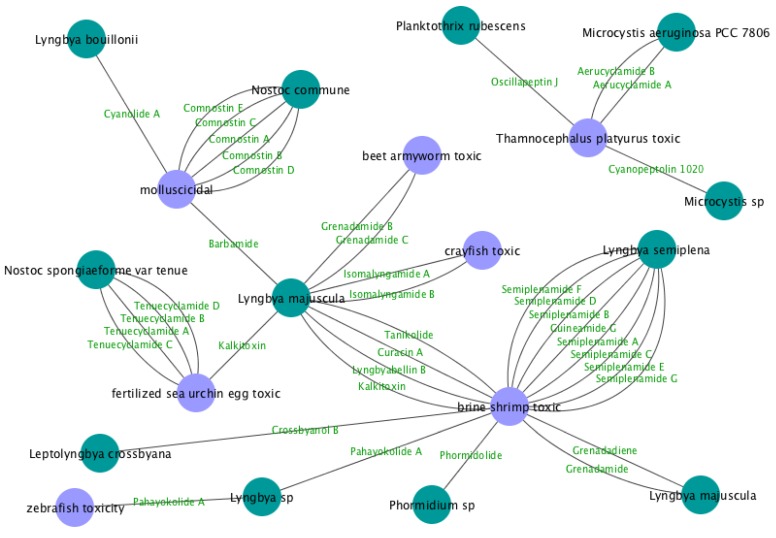
Interaction network of 36 compounds isolated from cyanobacteria that have been demonstrated to be toxic to invertebrates and have the potential to be developed as bio-molluscicide/bio-ovicide agents.

This query was limited to articles published before 31/03/2014. This yielded a total of 3430 documents, curation of which allowed for the identification of 36 toxicity-inducing chemical compounds (Figure 1, Table 1) that have potential to be developed as bio-molluscicide/bio-ovicide agents.

**Table 1:** Invertebrate toxicity-inducing compounds isolated from cyanobacteria.

Compound	Species	Biological Activity	References
Grenadamide B	*Lyngbya majuscula*	beet armyworm toxic	[6]
Grenadamide C	*Lyngbya majuscula*	beet armyworm toxic	[6]
Crossbyanol B	*Leptolyngbya crossbyana*	brine shrimp toxic	[7]
Curacin A	*Lyngbya majuscula*	brine shrimp toxic	[8]
Grenadadiene	*Lyngbya majuscula*	brine shrimp toxic	[9]
Grenadamide	*Lyngbya majuscula*	brine shrimp toxic	[9]
Guineamide G	*Lyngbya semiplena*	brine shrimp toxic	[10]
Lyngbyabellin B	*Lyngbya majuscula*	brine shrimp toxic	[11]
Phormidolide	*Phormidium* sp.	brine shrimp toxic	[12]
Semiplenamide A	*Lyngbya semiplena*	brine shrimp toxic	[13]
Semiplenamide B	*Lyngbya semiplena*	brine shrimp toxic	[13]
Semiplenamide C	*Lyngbya semiplena*	brine shrimp toxic	[13]
Semiplenamide D	*Lyngbya semiplena*	brine shrimp toxic	[13]
Semiplenamide E	*Lyngbya semiplena*	brine shrimp toxic	[13]
Semiplenamide F	*Lyngbya semiplena*	brine shrimp toxic	[13]
Semiplenamide G	*Lyngbya semiplena*	brine shrimp toxic	[13]
Tanikolide	*Lyngbya majuscula*	brine shrimp toxic	[14]
Kalkitoxin	*Lyngbya majuscula*	brine shrimp toxic; fertilized sea urchin egg toxic	[15]
Pahayokolide A	*Lyngbya* sp.	brine shrimp toxic; zebrafish toxicity	[16]
Isomalyngamide A	*Lyngbya majuscula*	crayfish toxic	[17]
Isomalyngamide B	*Lyngbya majuscula*	crayfish toxic	[17]
Tenuecyclamide A	*Nostoc spongiaeforme var. tenue*	fertilized sea urchin egg toxic	[18]
Tenuecyclamide B	*Nostoc spongiaeforme var. tenue*	fertilized sea urchin egg toxic	[18]
Tenuecyclamide C	*Nostoc spongiaeforme var. tenue*	fertilized sea urchin egg toxic	[18]
Tenuecyclamide D	*Nostoc spongiaeforme var. tenue*	fertilized sea urchin egg toxic	[18]
Barbamide	*Lyngbya majuscula*	molluscicidal	[19]
Comnostin A	*Nostoc commune*	molluscicidal	[20]
Comnostin B	*Nostoc commune*	molluscicidal	[20]
Comnostin C	*Nostoc commune*	molluscicidal	[20]
Comnostin D	*Nostoc commune*	molluscicidal	[20]
Comnostin E	*Nostoc commune*	molluscicidal	[20]
Cyanolide A	*Lyngbya bouillonii*	molluscicidal	[21]
Aerucyclamide A	*Microcystis aeruginosa PCC 7806*	Thamnocephalus platyurus toxic	[22]
Aerucyclamide B	*Microcystis aeruginosa PCC 7806*	Thamnocephalus platyurus toxic	[22]
Cyanopeptolin 1020	*Microcystis* sp.	Thamnocephalus platyurus toxic	[23]
Oscillapeptin J	*Planktothrix rubescens*	Thamnocephalus platyurus toxic	[24]

## 2. Invertebrate Toxicity Related Research

The search for compounds specifically toxic to invertebrates such as molluscs are of importance for the agricultural industry, as well as for use as agents that will limit transmission of trematode parasite-derived diseases via their intermediate mollusc host that affect both human health and animal health [25,26,27]. The control of these diseases can be achieved by limiting the intermediate host. Synthetic Niclosamide (NCL) is currently the only molluscicide recommended by the World Health Organization (WHO). Its use, is however, environmentally hazardous as it is toxic to fish at effective molluscicidal concentrations, thereby causing loss where fishing is an important economic activity or food source [28].

Whole animal bioassays (sea urchin, brine shrimp, beavertail fairy shrimp and crayfish toxicity bio-assays) are frequently used to evaluate biological effects of pollutants on marine organisms and for the preliminary assessment of natural marine products for industrial and pharmaceutical activities of interest. The objective of such assays is to detect toxic effects on representative of a given ecosystem. Carballo *et al.* (2002) [29] evaluated the suitability of the brine shrimp lethality assay and the inhibition of hatching of cyst assays to test natural marine products from 14 species of marine invertebrates and 6 species of macroalgae for pharmacological activity. They demonstrated that the invertebrate extracts were the most toxic and that some species of echinoderms, the sponges *Mycale parishii*, *Dysidea* sp. and the gorgonians *Pacifigorgia adamsii Muricea* sp. significantly lowered hatching in the hatchability test, interfering with normal development of the *Artemia nauplii* and that this toxicity correspond to cytotoxicity in 50% of samples tested against two human cell lines, lung carcinoma A-549 and colon carcinoma HT-29 [29]. Thus, some of these extracts may be toxic to invertebrates only. Moreover, sea urchin egg bioassay has additionally been used as a model for evaluation of developmental toxicology, as well as the brine shrimp bioassay based on the inhibition of hatching of cyst [30,31,32]. These bioassays are useful tools to identify potential bio-ovicide chemical compounds.

## 3. Compounds that Exhibit Toxicity against Invertebrates

### 3.1. Compounds Toxic to Molluscs

**Figure 2:**
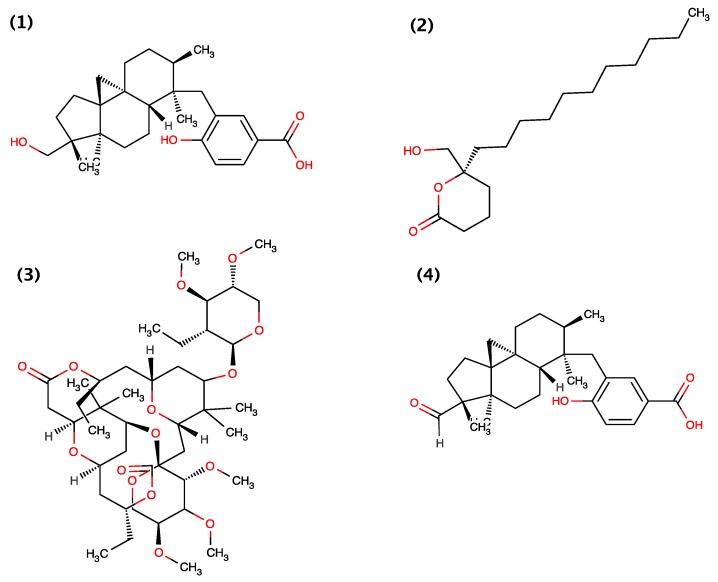
Structure of Barbamide (1), Tanikolide (2), Cyanolide A (3) and Comnostin B (4).

To date, only four compounds isolated from cyanobacteria have been shown to exhibit molluscicidal activity these include Barbamide, Tanikolide, Cyanolide A and Comnostin B (Figure 2). Barbamide, a chlorinated lipopeptide, was originally isolated from cyanobacterium *Lyngbya majuscule* belonging to the family *Oscillatoriaceae* [19]. Orjala and Gerwick (1996) demonstrated that Barbamide exhibits molluscicidal activity (absolute lethal concentration (LC_100_) = 10 μg/mL) against *Biomphalaria glabrata.* Later, the Barbamide biosynthetic gene cluster was characterized [33,34] and obtained from marine cyanobacterium *Moorea producens* and heterologously expressed in *Streptomyces venezuelae* that resulted in the production of a new barbamide congener 4-*O*-demethylbarbamide [35]. Ilardi and Zakarian (2011) [36] further demonstrated the total chemical synthesis of Barbamide.

Later, Singh *et al.* (1999) [14] isolated Tankolide from cyanobacterium *Lyngbya* *majuscula* collected from Madagascar. They demonstrated that Tanikolide exhibit molluscicidal activity against *Biomphalaria* *glabrata* with a median lethal dosage (LD_50_) of 9.0 μg/mL, brine shrimp toxicity against *Artemia* *salina* with a LD_50_ of 3.6 μg/mL and anti-fungal activity against *Candida* *albicans* giving a 13-mm zone of inhibition at 100 μg/disk. They additionally demonstrated that Tanikolide does not exhibit ichthyotoxicity against *Carassius* *auratus*; however, a narcotic effect was observed at 10 μg/mL [14]. Since then, [37,38,39,40,41] demonstrated the chemical synthesis of Tanikolide.

Jaki *et al.* (2000) [20] isolated five diterpenoid extracellular metabolites, Comnostins A–E, (Figure 2) from *Nostoc commune* (EAWAG 122b) but demonstrated that only Comnostin B exhibit moluscicidal activity against *Biomphalaria glabrata* with a minimum inhibitory concentration (MIC) of 20 μg/mL and cytotoxicity in human cervical cancer (HeLa) subline KB cells and human caucasian colon adenocarcinoma (Caco-2) cells. Comnostin A–E were however shown to exhibit moderate antibacterial activity against *Bacillus cerius.* Comnostin C exhibited selectively potent antibacterial activity with a MIC value for *Escherichia coli* equal to that of tetracycline, and similarly for Comnostin E with a MIC value for *Staphylococcus epidermidis* equal to that of chloramphenicol [20]. Comnostins A, C, D and E have not been assessed for molluscicidal activity.

Alban *et al.* (2010) [21] isolated Cyanolide A (Figure 2), a novel glycosidic macrolide from the Papua New Guinea cyanobacterium *Lyngbya bouillonii.* They demonstrated that Cyanolide A exhibited potent molluscicidal activity against *Biomphalaria glabrata* with a median lethal concentration (LC_50_) of 1.2 μM [21]. Since then, [42,43,44,45,46,47,48,49] demonstrated the chemical synthesis of Cyanolide A.

### 3.2. Compounds Toxic to Beet Armyworms

**Figure 3:**
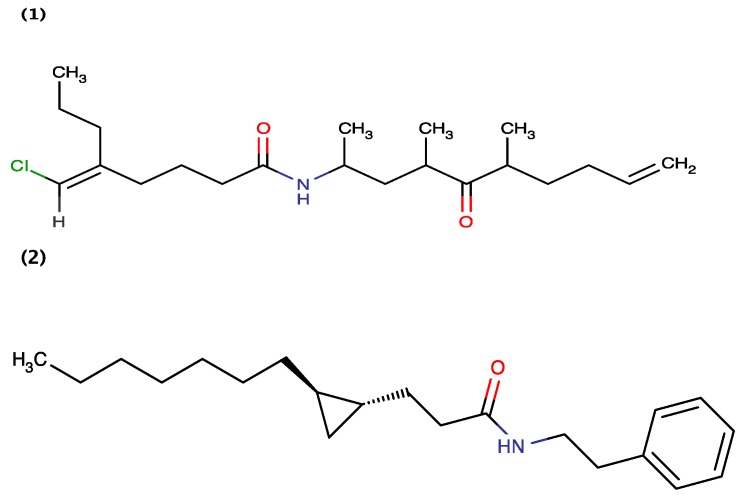
Structure of Grenadamide B (1) and Grenadamide C (2).

Jiménez *et al.* (2009) [6] isolated Grenadamides B and C (Figure 3) from the Caribbean cyanobacterium *Lyngbya majuscule.* It was demonstrated that 1 mg/mL of Grenadamides B and C exhibits 38% and 50% mortality, respectively, for the insecticidal assay against beet armyworms [6]. Grenadamide A has not assessed for toxicity towards beet armyworm, but was shown to exhibit toxicity towards brine shrimp (discussed below).

### 3.3. Compounds Toxic to Fertilized Sea Urchin Eggs

**Figure 4:**
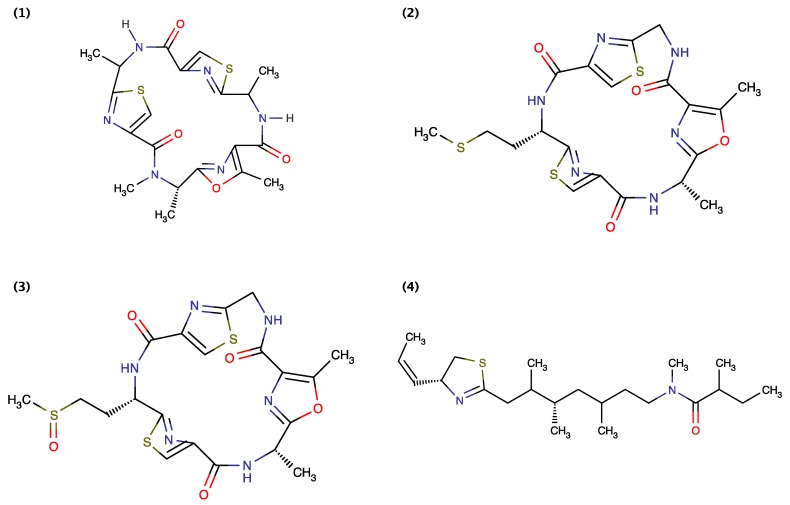
Structure of Tenuecyclamide A (1), Tenuecyclamide C (2), Tenuecyclamide D (3) and Kalkitoxin (4).

Tenuecyclamides A–D (Figure 4) and known antibiotic Borophycin were extracted from *Nostoc spongiaeforme* var*. tenue* (TAU strain IL-184-6) isolated from a litophytic sample collected in the Volcani Center, Bet Dagan, Israel [18]. Banker and Carmeli (1998) demonstrated that Tenuecyclamide A, C and D inhibit the division of sea urchin embryos with an effective dose (ED_100_) value of 10.8 μM, 9.0 μM, and 19.1 μM, respectively. Sea urchin egg toxicity exhibited by Tenuecyclamide B was not assessed [18]. Wu *et al.* (2000) [15] used a combination of brine shrimp and fish toxicity assays for the bioactivity-guided isolation of Kalkitoxin from the Caribbean cyanobacterium *Lyngbya majuscule.* White *et al.* (2004) [50] further demonstrated that Kalkitoxin also exhibits cytotoxicity to the human colon carcinoma cell line HCT-116 with a half maximal inhibitory concentration (IC_50_) value of 1.0 μg/mL. LePage demonstrated that Kalkitoxin blocks neurotoxicity in cerebellar granule neuron cultures (CGN) induced with 30 mM veratridine, with half of the maximal effective concentration (EC_50_) value of 22.7 nM [51]. Also, the chemical synthesis of Kalkitoxin was demonstrated by [52,53].

### 3.4. Compounds Toxic to Brine Shrimp

**Figure 5:**
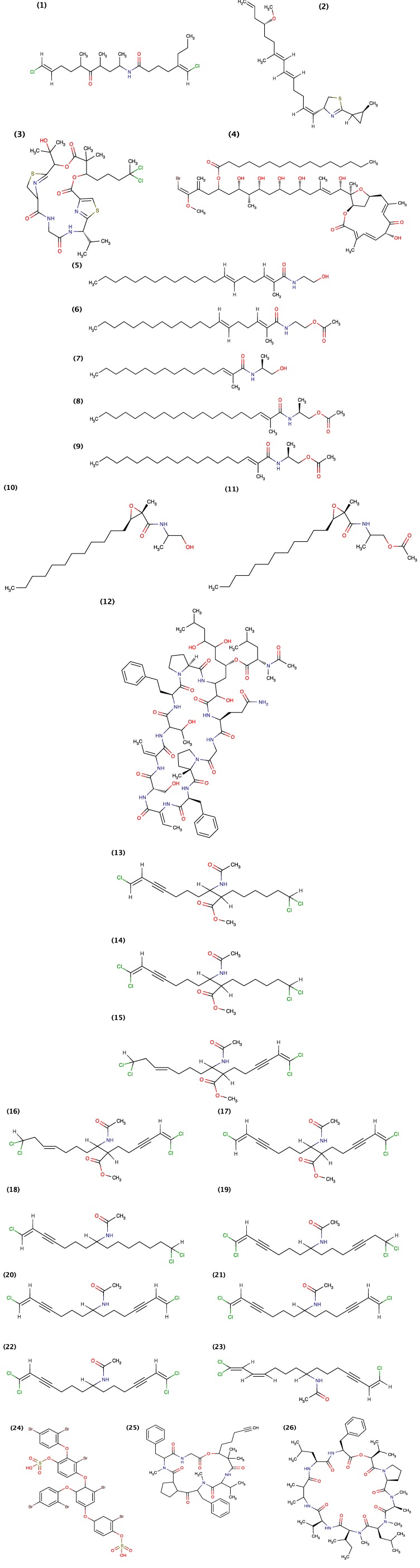
Structure of Curacin A (1), Grenadamide A (2), Lyngbyabellin B (3), Phormidolide (4), Semiplenamide A (5), Semiplenamide B (6), Semiplenamide C (7), Semiplenamide D (8), Semiplenamide E (9), Semiplenamide F (10), Semiplenamide G (11), Pahayokolide A (12), Taveuniamides A (13), Taveuniamides B (14), Taveuniamides C (15), Taveuniamides D (16), Taveuniamides E (17), Taveuniamides F (18), Taveuniamides G (19), Taveuniamides H (20), Taveuniamides I (21), Taveuniamides J (22), Taveuniamides K (23), Crossbyanol B (24), Guineamide G (25) and Wewakamide A (26).

Gerwick *et al.* (1994) [8] used the brine shrimp assay for the bioactivity-guided isolation of Curacin A (Figure 5) from the Caribbean cyanobacterium *Lyngbya majuscula*. They demonstrated that Curacin A exhibits brine shrimp toxicity with an IC_50_ value of 3 ng/mL. They additionally demonstrated that Curacin A exhibits antiproliferative activity with an IC_50_ value of 6.8 ng/mL in the Chinese hamster Aux B1 cell line, as well as antimitotic activity that inhibits microtubule assembly and the binding of colchicine to tubulin with an IC_50_ values in L1210 Leukemia and CA46 Burkitt Lymphoma cell lines ranging from 7 to 200 nM [8]. Yoo and Gerwick [54] isolated Curacins B and C and Marquez *et al.* [55] isolated Curacin D from same cyanobacterium. Curacins B, C and D were also demonstrated to exhibit antimitotic activity, but for these compounds toxicity to brine shrimp was not accessed. Synthesis of Curacin A is reported in [56], while [57,58,59,60,61,62,63] have contributed to our understanding of Curacin A biosynthesis. Later, Sitachitta and Gerwick (1998) isolated cyanobacterium *Lyngbya majuscula* from a shallow water (4−6 m) sample collected from Grand Anse Beach, Grenada. From this cyanobacterium they extracted cyclopropyl-containing fatty acid derivatives that include Grenadadiene, Debromogrenadiene and Grenadamide A and demonstrated that the metabolites have different bioactive profile. Grenadamide A exhibited toxicity towards brine shrimp with an LD_50_ value of 5 μg/mL and cannabinoid receptor binding activity with a absolute inhibition constant (*K*_i_) value of 4.7 μM, and Grenadadiene exhibits cytotoxicity against the NCI 60 cell line. In contrast, Debromogrenadiene exhibited no bioactivity in these assays. Milligan *et al.* (2000) [11] and Luesch *et al.* (2000) [64] isolated Lyngbyabellin B from cyanobacterium *Lyngbya majuscula* collected from an area near the Dry Tortugas National Park, Florida, and Apra Harbor, Guam, respectively. Milligan *et al.* demonstrated that Lyngbyabellin B exhibits toxicity toward brine shrimp with an LD_50_ value of 3 ug/mL and the fungus *Candida albicans* giving a 10.5-mm zone of inhibition at 100 μg/disk [11]. Luesch *et al.* further demonstrated that Lyngbyabellin B also exhibits cytotoxicity against KB and human colon adenocarcinoma (LoVo) cell line with IC_50_ values of 0.10 and 0.83 μg/mL, respectively [64]. In 2002, Ras-Raf protein interaction assay was used for the bioactivity-guided isolation of macrolide, Phormidolide from marine cyanobacterium *Phormidium* sp. [12]. Williamson *et al.* (2002) [12] demonstrated that Phormidolide exhibited toxicity towards brine shrimp with an LC_50_ value of 1.5 mM. Han *et al.* (2003) [13] also reported the extracted Semiplenamides A–G from the Papua New Guinea cyanobacterium *Lyngbya semiplena*. They demonstrated that Semiplenamides A–G exhibits toxicity against brine shrimp with LD_50_ values of 1.4, 2.5, 1.5, 18, 19, 1.4, and 2.4 μM, respectively. They also demonstrated that semiplenamide A inhibits the anandamide membrane transporter (AMT) with an IC_50_ value of 18.1 uM and semiplenamides A, B, and G showed affinity for the rat cannabinoid CB_1_ receptor where *K*_i_ values were 19.5 ± 7.8, 18.7 ± 4.6, and 17.9 ± 5.2, respectively [13]. Berry *et al.* (2004) [14] isolated Pahayokolide A from cyanobacterium *Lyngbya* sp. strain 15–2 collected from the floating periphyton mat in the Florida Everglades. They demonstrated that Pahayokolide A exhibited marginal toxicity to brine shrimp *Artemia* *salina* at the highest concentration tested (1 mg/mL) and toxicity to zebrefish (*Danio rerio*) embryos activity with LC_50_ of 2.25 μM. They additionally demonstrated that Pahayokolide A inhibited representatives of *Bacillus* (*B. megaterium* and *B. subtilis*), cyanobacteria (*Nostoc* Ev-1), green algae (*Ulothrix* Ev-17, *Chlamydomonas* Ev-29 and *Chlorella* 2–4) and yeast (*Saccharomyces cerevisiae*) as well as exhibits toxicity towards numerous cancer cell lines [14]. Since then, [16] and [65] contributed to the structure elucidation of Pahayokolide A and B. Williamson *et al.* (2004) [66] also used the brine shrimp assay for the bioactivity-guided isolation of chlorinated lipids, Taveuniamides A–K from two mixed Fijian collections of the cyanobacteria *Lyngbya majuscula* and *Schizothrix* sp. They demonstrated that Taveuniamide F, G and K were the most potent toxins against brine shrimp with LD_50_ values ranging between 1.7–1.9 μg/mL [66]. Taveuniamides A–K have not been accessed for other toxicities. Choi *et al.* (2010) [7] isolated four heptabrominated polyphenolic ethers, Crossbyanols A−D from the Hawaiian coral reef cyanobacterium *Leptolyngbya crossbyana*. Crossbyanol B uniquely exhibted potent toxicity against brine shrimp with an IC_50_ value of 2.8 μg/mL and also showed the most potent antibacterial activity with an MIC value of 2.0−3.9 μg/mL against methicillin-resistant *Staphylococcus aureus* (MRSA). Crossbyanol A displayed a different bioactivity profile compared to Crossbyanol B, as Crossbyanol A displayed weak cytotoxicity against human lung cancer cells (H-460) with an IC_50_ value of 30 μg/mL and activated voltage-gated sodium channel in mouse brain neuroblast cells (Neuro-2a) with an IC_50_ value of 20 μg/mL. Crossbyanols C and D were not active in these assays at a maximum test concentration of 20 μg/mL [7]. Han *et al.* (2011) [10] isolated two novel cyclic depsipeptides, Guineamide G and Wewakamide A from the Papua New Guinea cyanobacteria *Lyngbya majuscula* and *Lyngbya semiplena*, respectively. Both Guineamide G and Wewakamide A exhibited potent toxicity against brine shrimp, while only Guineamide G exhibited cytotoxicity towards mouse neuroblastoma cells with LC_50_ values of 2.7 μM [10]. Guineamides A–F, also isolated from Papua New Guinea cyanobacteria *Lyngbya majuscule*, were not evaluated for toxicity against brine shrimp [67].

### 3.5. Compounds Toxic to Beavertail Fairy Shrimp

**Figure 6:**
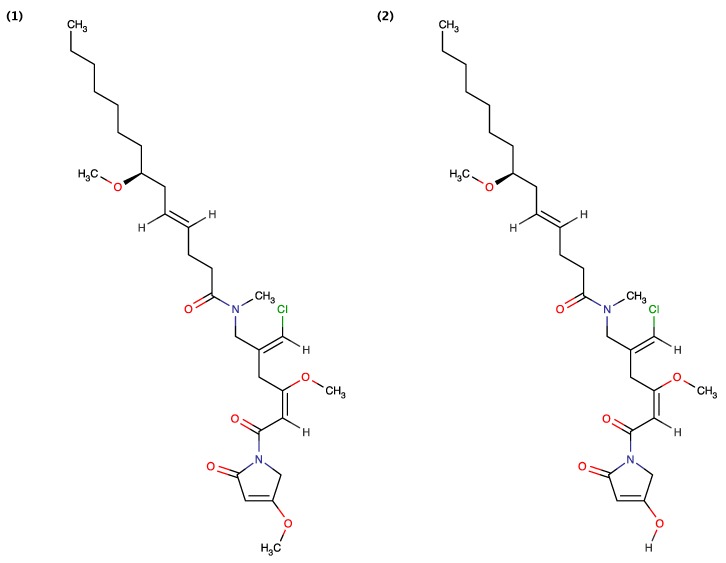
Structure of Aerucyclamide A (1), Aerucyclamide B (2), Cyanopeptolin 1020 (3) and Oscillapeptin J (4).

Blom *et al.* (2003) [24] used the freshwater crustacean *Thamnocephalus platyurus* toxicity assay for the bioactivity-guided isolation of Oscillapeptin J (Figure 6) from cyanobacterium *Planktothrix rubescens*, an axenic isolate from Lake Zürich. They demonstrated that Oscillapeptin J exhibited toxicity against *Thamnocephalus platyurus* with a LC_50_ value of 15.6 μM [24]. Portmann *et al.* (2008) [22] extracted Aerucyclamides A and B from the toxic freshwater cyanobacterium *Microcystis aeruginosa* PCC 7806 isolated from a Pasteur Culture Collection of Cyanobacteria in Paris, France. They demonstrated that Aerucyclamides A and B are toxic to the freshwater crustacean *Thamnocephalus platyurus* with LD_50_ values of 30.5 and 33.8 mM, respectively. Portmann *et al.* further demonstrated that synthetic Aerucyclamide B could be obtained through oxidation of Aerucyclamide A (MnO_2_, benzene) [22]. Later, Cyanopeptolin 1020 was extracted from the *Microcystis aeruginosa* strain UV-006 isolated from the Hartebeespoort Dam near Pretoria, South Africa [23]. Gademann *et al.* (2010) [23] demonstrated that Cyanopeptolin 1020 is toxic to the freshwater crustacean *Thamnocephalus platyurus* with LC_50_ values of 8.8 μM. They additionally reported IC_50_ values for factor XIa (3.9 nM), kallikrein (4.5 nM), plasmin (0.49 μM) and chymotrypsin (1.8 μM) and that Cyanopeptolin 1020 did not display inhibitory properties against the human plasminogen activator, thrombin, and human LMW (low molecular weight) urokinase at concentrations below 2.5 μM [23].

### 3.6. Compounds Toxic to Crayfish

**Figure 7:**
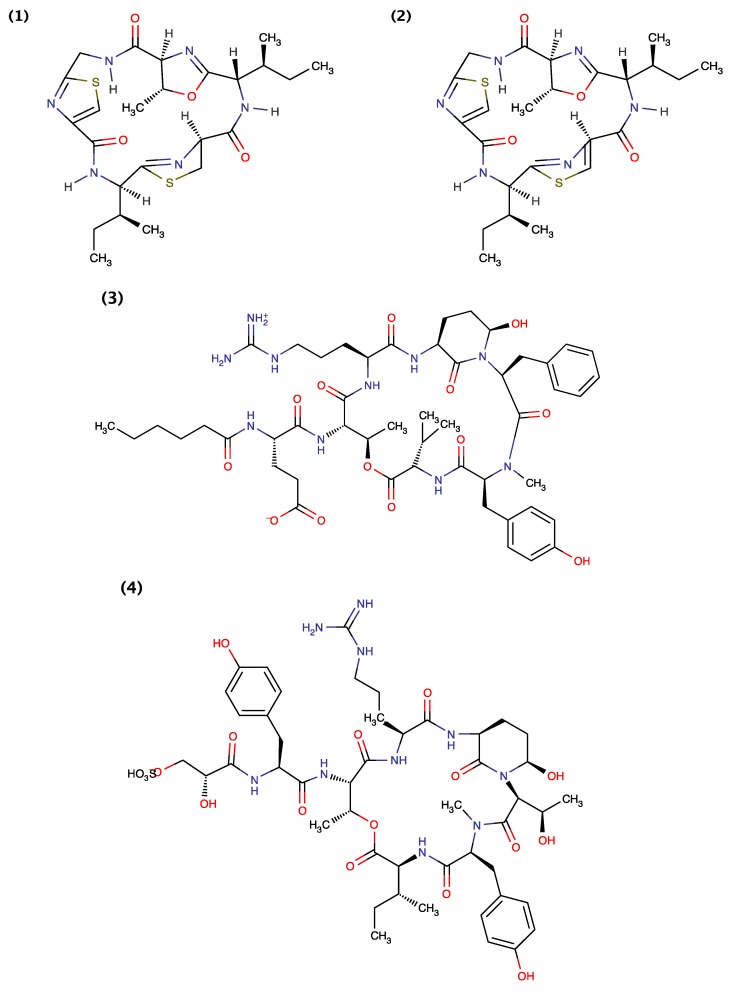
Structure of Isomalyngamide A (1) and Isomalyngamide B (2).

Isomalyngamides A and B (Figure 7) were extracted from the Hawaiian cyanobacterium *Lyngbya majuscule* [17]*.* Kan *et al.* (2000) [17] demonstrated that Isomalyngamides A and B are toxic to crayfish *Procambarus clarkii* by intraperitoneal injection at 250 and 500 μg/kg, respectively. Tan *et al.* (2010) [68] later demonstrated that Isomalyngamide A is a potential antifoulant that exhibits anti-larval settlement activity against cyprid larvae of the barnacle, *Amphibalanus amphitrite* (previously *Balanus amphitrite*) with an EC_50_ value of 2.6 μg/mL. Isomalyngamides A displayed low toxicity (<15%) but significant anti-settlement activities with >80% of the barnacle cyprids found swimming (*i.e.*, unsettled) when compared with the controls [68]. Chang *et al.* (2011) [69] and More *et al.* (2013) [70] demonstrated that Isomalyngamides A suppress metastatic events (e.g., migration, invasion and adhesion) in both the human breast carcinoma MCF-7 and human breast adenocarcinoma MDA-MB-231 cell line at “nontoxic” concentration [69,70].

## 4. Conclusions

Despite cyanobacteria being identified for their promising application as a renewable source for many industrially desired compounds, the evaluation of compounds isolated from cyanobacteria for molluscicidal activity has not received much attention. Moreover, the preliminary criterion for chemical compounds being identified, as potential pesticides should be broadened to include all invertebrate-targeting biological agents, with both terrestrial and aquatic invertebrates, instead of mollusc-targeting biological agents exclusively. In this manner, a more appropriate invertebrate-targeting biological agent similar to the iron EDTA complex, which may be an attractive feed for both mature and juvenile snails and an effective bio-ovicide, can be unearthed. Furthermore, broadening the preliminary criterion for the identification of the potential moluscicide may bring to light chemical compounds that have the potential to control other emerging invertebrate pests including armyworms, earwigs, millipedes, weevils and rutherglen bugs. Effectiveness of a pesticide is evaluated based on degradability and accumulation characteristic, as well as selective toxicity. However, this data is not available for the chemical compounds collated in this review. We therefore hope that this review will serve as a foundation that will promote research on the topic of this study, through which this missing information will also be obtained.
